# Interplay between the Hepatitis B Virus and Innate Immunity: From an Understanding to the Development of Therapeutic Concepts

**DOI:** 10.3390/v9050095

**Published:** 2017-04-28

**Authors:** Suzanne Faure-Dupuy, Julie Lucifora, David Durantel

**Affiliations:** Cancer Research Center of Lyon (CRCL), INSERM, U1052, CNRS, University of Lyon, UMR-5286, 69003 Lyon, France; suzanne.faure-dupuy@inserm.fr (S.F.-D.); julie.lucifora@inserm.fr (J.L.)

**Keywords:** HBV, liver immunity, innate immunity, viral escape, immune therapeutic concepts

## Abstract

The hepatitis B virus (HBV) infects hepatocytes, which are the main cell type composing a human liver. However, the liver is enriched with immune cells, particularly innate cells (e.g., myeloid cells, natural killer and natural killer T-cells (NK/NKT), dendritic cells (DCs)), in resting condition. Hence, the study of the interaction between HBV and innate immune cells is instrumental to: (1) better understand the conditions of establishment and maintenance of HBV infections in this secondary lymphoid organ; (2) define the role of these innate immune cells in treatment failure and pathogenesis; and (3) design novel immune-therapeutic concepts based on the activation/restoration of innate cell functions and/or innate effectors. This review will summarize and discuss the current knowledge we have on this interplay between HBV and liver innate immunity.

## 1. Role of Liver Innate Immunity in Pathogen Clearance and Immune-Driven Pathogenesis: Generalities

The liver, which is made up of approximately 80% hepatocytes, is often considered a secondary lymphoid organ due to the amount of flowing-through, infiltrating, and resident immune cells it contains [1,2]. Notably, innate immune cells, as well as non-parenchymal/non-professional cells endowed with innate functions (e.g., liver sinusoidal endothelial cells (LSEC), hepatic stellate cells (HSC)) [3], are particularly enriched in this solid organ. By order of decreasing abundance, innate cells from: (1) lymphoid origin (natural killer and natural killer T-cells (NK/NKT) but also innate lymphoid cells (ILC), and mucosal-associated invariant T-cell (MAIT)); (2) myeloid origin (e.g., Kupffer cells (KCs), myeloid-derived suppressive cells (MDSC), monocytes, neutrophils); and (3) various types of dendritic cells (DCs; mDC-BDCA1+, mDC-BDCA3+, plasmacytoid DCs; pDCs), can be found in a healthy liver. These cells, as well as the cytokinic/chemokinic effectors that they produce, can be beneficially involved in the early containment of pathogen infection and the orchestration of pathogen-specific adaptive responses [1,3]. However, they can also be involved in immune-driven pathogenesis, if a return to homeostasis is impaired by a pathogen or a recurrent exposure to xenobiotics/alcohol [4,5,6]. With respect to the latter, sterile or pathogen-driven chronic inflammation, as well as non-specific and uncontrolled cytotoxic activities, can be associated with the development of steatohepatitis, fibrosis, cirrhosis, and hepatocellular carcinoma [4,5,6].

Many liver innate immune cells are involved in the so-called tolerogenic microenvironment, which prevails and protects this organ against permanent exposure to gut-derived microbial degradation products or a low concentration of living bacteria [7]. Incidentally, the liver houses many chronic infections, including type B and C viral hepatitis, as well as parasitic infections [3]. Despite this intrinsic immune tolerance, the liver is well equipped to mount a potent antimicrobial response, and successful pathogens have also evolved strategies to either passively or actively evade innate and adaptive immune responses in order to persist.

For the hepatitis B virus (HBV), it has been clearly shown that adaptive responses are needed for an efficient and persistent control of infection [8,9]. However, the role of innate immunity has often been overlooked, as HBV infection is usually diagnosed several weeks after the onset of infection, when the virus has already escaped from early immune responses and viremia is high [10,11]. For this reason, the role of innate immune cells and their effectors, in HBV persistence and associated-pathogenesis, has yet to be actively investigated. This review will discuss our current knowledge of the interplay between HBV and innate immune cells/effectors, as well as envisaged strategies to develop immune therapeutic concepts, particularly involving innate cells/effectors, and to achieve a functional cure (i.e., loss of the HBV surface antigen (HBsAg), with or without anti-HBs seroconversion).

## 2. Interplay between HBV and Innate Immunity: Basic Insights

### 2.1. HBV Recognition by Innate Sensors

The early and non-specific detection of pathogens generally occurs, at subcellular/molecular levels, via the recognition of Pathogen-Associated Molecular Patterns (PAMP) by innate immunity sensors, also called Pathogen Recognition Receptors (PRR). Amongst PRR, there are toll-like (TLR), Retinoic acid-Inducible Gene I (RIG)-like, nucleotide-binding oligomerization domain-containing protein (NOD)-like, C-type Lectin, and DNA-sensing receptors, which are differentially or ubiquitously expressed in various types of epithelial/endothelial cells, as well as professional and non-professional immune cells [12].

**Figure 1:**
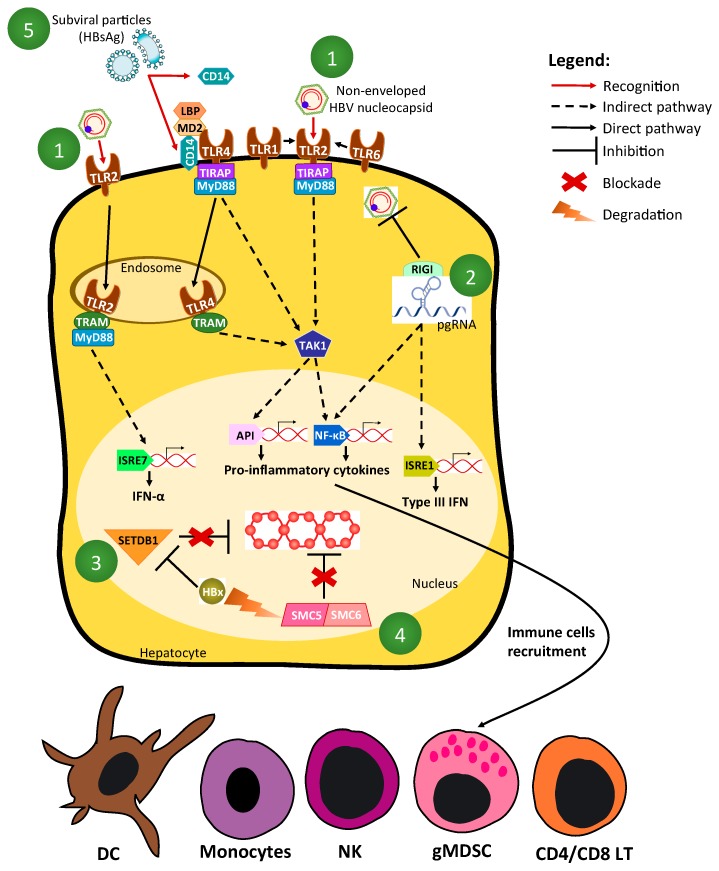
Hepatitis B virus (HBV) detection by innate immune sensors and regulation by host factors. (**1**) Non-enveloped HBV nucleocapsid composed of HBV core/capsid antigen (HBcAg) can be recognized by Toll-Like Receptor 2 (TLR2), triggering pro-inflammatory cytokine secretion in TLR2-positive liver cells via TRIF-Related Adaptor Molecule/ Myeloid Differentiation primary response gene 88 (TRAM/MyD88) or TIR domain-containing Adapter Protein (TIRAP)/MyD88 adaptation and Activator Protein 1 (AP1) and Nuclear Factor kappa-B (NFκB)-dependent pathways; (**2**) RIG-I could be a sensor of the “epsilon (ε) stem-loop” present in pregenomic RNA (pgRNA). This recognition leads to the production of type-III interferon (IFN) via both interferon regulatory transcription factor (IRF) 3 and NFκB-dependent pathways, as well as to the sequestration of pgRNA and a subsequent decrease of relaxed circular DNA (rcDNA) synthesis; (**3**) SETDB1, a histone-methyl-transferase, impairs covalently closed circular DNA (cccDNA) transcription, which can be reverted by HBx; (**4**) Structural Maintenance of Chromosome 5 and 6 (SMC5/6) complex inhibition of cccDNA transcription can be reverted by HBx, which induces SMC5/6 degradation in a DNA-Damage-Binding 1 (DDB1)-Cul4-E3-ubiquitin-ligase-dependent manner; (**5**) Subviral particles (i.e., HBV surface antigen or HBsAg) could interact with CD14, leading to their internalization and cell activation in TLR4 positive cells.

Upon interaction between a PRR and its cognate PAMP, various downstream signaling pathways are activated and sequentially involve: (1) adaptor/co-adaptor molecules (e.g., myeloid differentiation primary response gene 88 (MyD88), TIR-domain-containing adapter-inducing interferon-β (TRIF), etc.); (2) kinases (e.g., TANK binding kinase 1 (TBK1), transforming growth factor β-activated kinase 1 (TAK1), etc.) and (3) transcription factors (e.g., interferon regulatory transcription factors (IRFs), nuclear factor kappa-B (NFκB), c-fos/c-jun) [12] (Figure 1). This leads to the expression of effector genes, including interferon-stimulated-genes (ISG) and NF-κB-inducible or pro-inflammatory genes. Primary effectors include the various interferons (IFN; IFNs-α (13 different alleles), IFN-β, IFNs-λ (four alleles), and IFN-γ, as well as pro-inflammatory cytokines/chemokines (e.g., interleukin (IL)-6, tumor necrosis factor (TNF-α) which collectively have direct or indirect anti-microbial actions, and/or are involved in the recruitment of innate and adaptive immune cells to the infected or endangered site (Figure 1).

Other innate detection systems rely on canonical or non-canonical inflammasomes [13,14]. They are induced by PAMP or Danger-Associated Molecular Patterns (DAMP) and lead to the activation of the Caspase-1, which in turn cleaves precursor molecules (e.g., pro-IL-1β and pro-IL-18) to liberate active IL-1β and IL-18 in the infected or endangered microenvironment. In normal/non-transformed hepatocytes, it has recently been shown that most PRR are expressed and functional [15,16,17], with the potential exception of the cyclic GMP-AMP Synthase/STimulator of Interferon Gene (cGAS/STING) axis [18], and the lack of inflammasome activities [19].

Whether HBV is genuinely detected by PRR upon very short exposure (i.e., contact between viral particles/incoming materials and PRR), or after the amplification of its genome and expression of its proteins during its replicative life-cycle in hepatocytes, is a matter of debate. One difficulty comes from the fact that in vitro models used to study HBV replication are suboptimal. Indeed, to obtain a strong and productive infection in primary human hepatocytes, 100 to 1000 virus-genome-equivalents/cell of recombinant HBV virions are needed [20], whereas, as a basis for comparison, a multiplicity of infection of <0.1 colony-forming-unit/cell is only needed to infect hepatoma cells with the hepatitis C virus (HCV). Moreover, for an unknown reason, HBV is not capable of spreading/diffusing into a monolayer of hepatocytes in vitro, thus indicating that there are impairments in virion egress or/and re-entry, which prevent the spreading, and consequently, the possibility of an increased exposure to PRR. Another problem relies on the fact that the “quality” of HBV inoculum used for in vitro experiments needs to be optimal to generate genuine results. Most researchers use HBV produced by HepG2.2.15 [21] or HepAD38 cells [22], in order to efficiently infect primary human hepatocytes (PHH) [23], differentiated HepaRG [20], or Na+-taurocholate-cotransporting-polypeptide (NTCP)-expressing hepatoma cells [24]. Various levels of non-enveloped nucleocapsids may contaminate these inocula (supplementary material in [25]) and be at the origin of artefactual results. Moreover, an HBV core/capsid antigen (HBcAg) synthesized in bacteria may be contaminated with lipopolysaccharide (LPS)-like ligands, thus further confusing the matter [26]. Nevertheless, it has been suggested that the HBV nucleocapsid is a ligand for TLR2 [27] and that TLR2-engagement by cognate ligand leads to the strong production of pro-inflammatory cytokines by hepatocytes and/or other TLR2-positive cells [16,25,27,28,29]. However, the existence of this non-enveloped HBcAg ligand in vivo is still a matter of debate. The use of HBV virions purified from a patient would guaranty the relevance of experiments; however, so far, there is no clear demonstration that they can be used to reproducibly and efficiently infect hepatocytes ex vivo/in vitro.

In contrast to other viral entities, HBV is a poor inducer of innate immunity because of the intrinsic characteristics of its life cycle [30]. The viral relaxed circular DNA (rcDNA), as well as single-stranded DNA (ssDNA) or linear double-stranded DNA (dsDNA; intermediates/side products of reverse transcription), are likely recognized as damaged, non-methylated, and non-self DNAs. In contrast, the other viral nucleic acids, including covalently-closed-circular DNA (cccDNA; i.e., the nuclear template of transcription) and various viral RNAs, are “host-like” molecules, and are therefore non-differentiable from “self”. Being synthesized within nucleocapsids, rcDNA is protected from a potential recognition by DNA sensors. One possible HBV PAMP could be the ternary complex formed by the pregenomic RNA (pgRNA) HBc and polymerase proteins [30]. In this respect, RIG-I was proposed as a sensor of the “Epsilon (ε) stem-loop” within pgRNA, leading to both a measurable production of type-III IFN in PHH, and a “sequestration of pgRNA” associated with an effector-independent antiviral decrease in rcDNA synthesis (i.e., intrinsic-restriction-like mechanism) [31].

If covalently closed circular DNA (cccDNA) ends up being a minichromosome-like structure, likely indistinguishable from self-DNA, it appears that its initial establishment from rcDNA, as well as its early transcription after the onset of infection, are modulated by host proteins, which could be defined as “intrinsic restriction factors”. Hence, it was shown that both the histone-methyl-transferase SETDB1 [32] and the complex SMC5/6 [33] are capable of impairing cccDNA transcription early-on after the onset of infection, and that HBx is needed to either counteract the action of the former [32] or induce the degradation of the latter in a DDB1-Cul4-E3-ubiquitin-ligase-dependent manner [33].

Besides the potential detection of the different HBV nucleic acids, it has also been reported that subviral particles could interact with CD14, a co-receptor of TLR4 in myeloid cells, via their phospholipid moiety [34]; this could lead to the internalization of HBsAg into these cells and their activation [35]. In fact, the soluble fraction of CD14, bound to HBsAg, could be responsible for this activation [36]. The fact that the lipid moiety of HBsAg subviral particles is instrumental for this activation represents an interesting finding. Indeed, the lipidomic of viral particles and its evolution during the natural course of infection in a human is completely unknown; potential changes in lipid composition, with the incorporation of “toxic lipid” (e.g., peroxydated lipid), could be associated with changes in the immune characteristics of HBV subviral particles.

To summarize, the molecular determinants of a potential recognition of HBV PAMPs by PRRs are still poorly defined and further studies are necessary. This understanding is instrumental in the context of the development of PRR agonists, which could be used in immune-therapeutic combinations.

### 2.2. Mechanisms of HBV-Driven Inhibition of Innate-Signaling Pathways (Figure 1 and Figure 2)

An acute exposure to/infection by HBV, in vitro, in an animal model and in a human, does not lead to a strong activation of IFN and pro-inflammatory responses. The first demonstration came from a microarray study performed with the biopsies of experimentally-infected chimpanzees, showing that, in comparison with HCV, the exponential increase in HBV viremia was not associated with a significant/measurable gene expression change in their liver, including innate immunity genes [37]. In agreement with this analysis, no (or a weak) seric production of IFNs and cytokines/chemokines was found in acutely HBV-infected patients (i.e., patients who consulted before clearing acute infection or progressing to chronic stage), as compared to what could be seen with similar patients infected by HIV or HCV [38]. A production of the immunosuppressive cytokine IL-10 was, however, evidenced during the viral load increase and was associated with a transient inhibition of NK functions [39]. This increased production of IL-10 correlates with virus replication, and was also evidenced during virus-induced flares occurring in long-term chronically infected patients [40]. This points toward a virus-mediated manipulation of cytokines/chemokines production in favor of immune suppression. 

When studied ex vivo/in vitro, the acute exposure of hepatocytes or other liver cell types to HBV is also not globally associated with a strong induction on IFNs and pro-inflammatory cytokines/chemokines. Differentiated HepaRG and PHH infected by “qualitatively relevant” recombinant HBV inoculum did not secrete a measurable amount of IL-6 or IFN-β, despite a very weak and transient activation of gene expression [25]. Similarly, KCs exposed to the virus only transiently released a very weak amount of IL-6 [41] or IL-1β [19], suggesting that HBV could be detected/sensed, but could also readily impair nascent responses. This potential lack of measurable events could either be due to: (1) the viral genotype used, as an HBV genotype-C led to a measurable secretion of IFN-λ by infected hepatocytes (in contrast to genotype D used in other studies [31]); or (2) to the cell cultivation conditions, as micro-patterned PHH on fibroblast feeder cells exposed to HBV showed a significant induction of type-III IFN gene expression [42].

The weak response or lack of IFN and/or pro-inflammatory responses, could also be due to viro-implemented active mechanisms. In this respect, the recently performed analyses in Chronic Hepatitis B (CHB) patient biopsies have shown that many genes of innate immunity were down-regulated when compared to control patients, and the magnitude of this inhibition was dependent upon the HBeAg status and seric levels of HBsAg [43]. Even if these results were only obtained at RNA levels, they confirm previous studies that had shown a down regulation, at the protein level, of innate sensors such as TLR2 [44] or TLR9 [45]. These results are in sharp contrast with what has been observed in the woodchuck model, in which an increase of the expression of the innate immunity gene was observed [46]. Moreover, in liver-humanized mice, which are immune-deficient, HBV infection led to a very weak increased expression of some innate immune genes, but not a significant decrease [47]. This emphasizes that animal models may not always be predictive of what happens in humans.

In contrast to HDV [47,48], HBV infections seem to be associated with a lack of induction or even a decreased expression of ISGs or other pro-inflammatory genes, thus pointing toward mechanisms of active inhibition. The first demonstration that HBV could inhibit PRR signaling pathways came from a study, which showed that both HBeAg and HBsAg could antagonize the antiviral action of PRR agonists in murine parenchymal and non-parenchymal cells [49]. Despite its interest, the results of this study were challenged due to the murine nature of the cell types used, as well as because of the apparent lack of specificity of the observed inhibitory phenotype (i.e., all viral forms led to the inhibitory phenotype) and the lack of insights on the underlying mechanism of action.

More recently, we have shown that HBV could be sensed by hepatocytes, leading to an early (i.e., between 2 h and 8 h post infection) and moderate elevation of some innate immunity gene expression [25]. However, we observed that this response was transient, and that incoming HBV virions were capable, in the absence of neo-synthesized viral proteins, of blocking the induction of IFN genes triggered in trans by cognate ligands of TLR3, RIGI, or Melanoma Differentiation-Associated protein 5 (MDA5). We then found that the core/capsid/HBc protein was responsible for this inhibitory phenotype through the recruitment of a histone-methyl-transferase, i.e., Enhancer of Zeste Homologue2 (EZH2), on targeted gene promoters, which was in turn capable of establishing repressive epigenetic marks to prevent their inducibility [50]. If the capacity of HBc to bind to synthetic dsDNA, the host genome, or cccDNA was already known [51,52,53], its ability to recruit epigenetic modifying enzymes and modulate gene expression is a novel discovery, and upgraded this otherwise structural protein to a potential innate immunity regulator in infected hepatocytes.

Other viral proteins have been suggested to interfere with innate signaling pathways in hepatocytes [54]. The two which are secreted, HBsAg and HBeAg, are thought to be involved in many immune evasion processes. As previously discussed, they are capable of impairing the activation of all TLR pathways [49,55]. However, regarding their potential role in the inhibition of intrahepatic signaling pathways, experimental data are less clear. It has been proposed that HBe or/and a related intracellular form called p22, could bind to the co-adaptor of Myd88, TIRAP (also called MAL2), and therefore interfere with the TLR2 signaling pathway [56]. More surprisingly, it has also been suggested that HBs could inhibit this pathway by a mechanism involving the inhibition of the c-Jun N-terminal protein Kinase (JNK), which in turn, would prevent IL-12 production [57]. The HBx protein has also been endowed with many biological activities, including the regulation of innate pathways. Hence, it has been proposed that HBx could inhibit the dsRNA-mediated IFN response by either competitively interacting with host-factors of these pathways (e.g., Mitochondrial Anti-Viral-Signaling protein (MAVS), TRIF, IRF3) and/or inducing their degradation by proteasome [58,59,60,61]. However, it is worth noting that the overexpression of this protein has often been associated with many un-relevant activities [62]. Finally, the HBV Pol has also been described to inhibit the dsRNA-mediated IFN response by interfering with STING and Dead box protein 3 (DDX3) functions [63,64]. 

To conclude, there is a lot of published data, however there are still many caveats in demonstrations and an overall lack of knowledge of underlying mechanisms. It seems appropriate to indicate that if the HBV-driven strategies to inhibit immune responses are genuine, they are only adapted to what HBV itself would be able to trigger as a weak inducer of innate responses; indeed, it seems that HBV-mediated inhibitory phenotypes are not very potent and are likely not capable of fully counteracting a more aggressive induction of innate responses, like that mediated, for instance, by HDV [48].

### 2.3. Modulation of Innate Cell Number/Frequency by HBV

One way for HBV to initially and persistently escape innate immune cell antiviral functions would be to modulate their numbers, ideally within the infected liver. There are no robust and convincing data showing that the number of CD14^+^ monocytes, resident KCs, pDC (BDCA2^+^ cells), mDC-BDCA3^+^, and NK/NKT cells within the infected liver, or in the peripheral blood, is affected by HBV throughout the natural history of infection [35,65,66,67,68,69]. 

**Figure 2:**
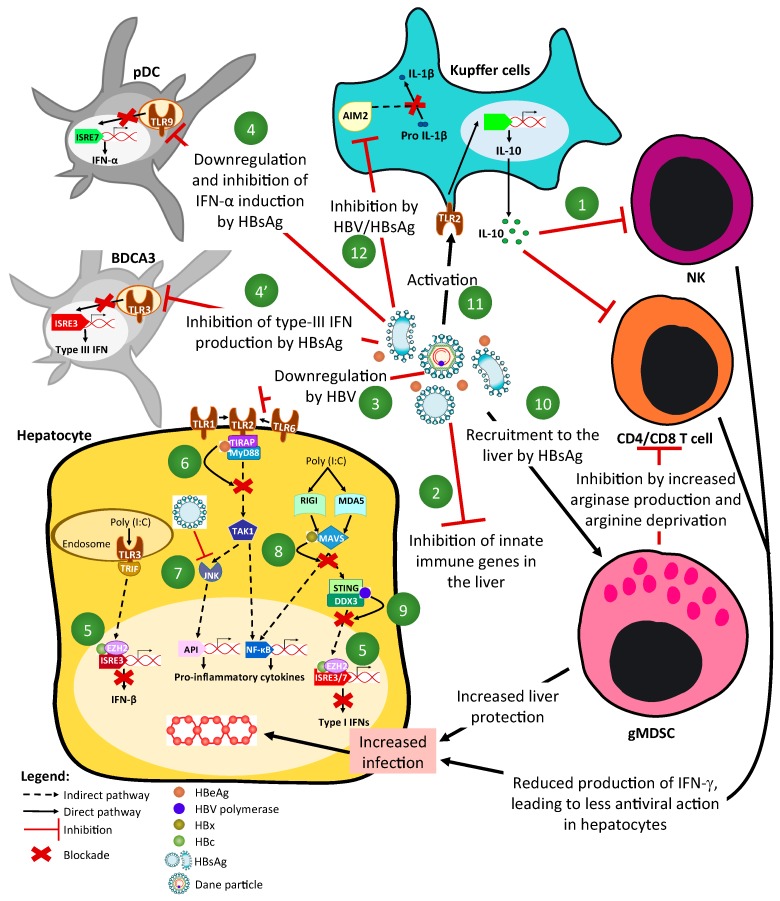
HBV modulation of innate immune sensors. (**1**) Interleukin-10 (IL-10) production can impair Natural Killer (NK) cell function, including the non-cytotoxic anti-HBV action of IFN-γ in infected hepatocytes; (**2**) HBV inhibit innate immune gene inductions in the liver according to HBeAg and HBsAg status; (**3**) TLR2 is down-regulated by HBV in hepatocytes, leading to a reduced production of pro-inflammatory cytokines/chemokines; (**4**) HBsAg down-regulates TLR9 in pDC, leading to the inhibition of IFN-α production and; (**4’**) HBsAg inhibits type-III IFN production upon TLR3 stimulation, hence preventing a relevant NK cell activation; (**5**) HBc can block the production of IFN upon dsRNA recognition receptor stimulation by the recruitment of EZH2 and immune promoters methylation; (**6**) HBeAg binds to TIRAP/MyD88 complex to inhibit the TLR2 signaling pathway; (**7**) HBsAg inhibits JNK pathway preventing IL-12 production; (**8**) HBx inhibits the dsRNA-mediated IFN response by interacting with host-factors (MAVS, TRIF, IRF3) and/or inducing their degradation by proteasome; (**9**) HBV polymerase inhibits the dsRNA-mediated IFN response by interfering with the STING and DDX3 function; (**10**) HBsAg can recruit and trigger the expansion of granulocytic Myeloid-Derived Suppressive Cells (gMDSC), which in turn, specifically inhibit CD4+ and CD8+ T-cell by the production of a large amount of arginase, leading to metabolic deprivation; (**11**) HBV can activate Kupffer cells (KCs) by the TLR2 pathway, leading to the production of IL-10 and the subsequent inhibition of the lymphocyte response; (**12**) HBsAg inhibits the AIM2-inflammasome and blocks the production of IL-1β, which has a strong antiviral effect on HBV replication in hepatocytes.

Therefore, HBV infections may instead be associated with the impairment of functions rather than the number/frequency of cells. There is at least one convincingly described exception to this, which is the number of myeloid-derived suppressive cells in CHB patients [70]. Indeed, it was recently reported that the number of granulocytic subsets of MDSC is increased in the low-inflammatory (immune tolerant) phase of the HBV natural history. Their number is inversely proportional to the level of liver inflammation, thus suggesting that these cells contribute to the protection of the liver, while likely favoring HBV replication. Although the mechanism has not been completely elucidated, HBV is likely to induce their recruitment/expansion in the liver to its own benefit. In the infected organ, MDSC inhibit HBV-specific CD4^+^ and CD8^+^ T-cell responses via metabolic means. Indeed, MDSC secrete a very high amount of arginase in the liver microenvironment, which in turn, decreases the amount of available arginine, an amino acid crucial for lymphocyte physiology and growth. This leads to their starving and functional inhibition. HBsAg could trigger their recruitment/expansion by acting through the ERK/Interleukin-6/Signal Transducer and Activator of Transcription 3 (ERK/IL-6/STAT3) signaling pathway [71] (Figure 2).

### 2.4. Modulation of Dendritic and NK/NKT Cells Functions by HBV (Figure 2)

More than affecting their numbers, HBV seems to impair the functions of many innate immune cells. An investigation on HBV-driven inhibitory phenotypes and underlying mechanisms is not easy in the absence of a good animal model and a low access to human liver samples. Moreover, studying liver phenotypes is more relevant than studying peripheral blood ones, as intimate contact/interaction between infected hepatocytes and immune cells can only take place within the liver. Unfortunately, most of the studies performed so far were with blood-derived cells, thus limiting their meaningfulness and feeding the debate around the “systemic perturbation” of innate functions by HBV.

pDCs are a unique dendritic cell (DC) subset specialized in IFN-α production, representing 10% of total DC in the liver (our unpublished data), and are very important antiviral innate cells [72]. Their number in CHB patients seems to be unchanged in both the blood and liver compartments [67,73]. However, it was shown that blood- and liver-derived pDC from CHB patients in an ex vivo study were less capable of producing IFN-α upon TLR9 agonisation [45,67,73], less capable of cross-talking and activating the cytolytic activity of naive NK cells [67], and more prone to induce the generation of regulatory T-cells [74]. HBsAg was suggested to be the main driver of these modulations, recapitulating these phenotypes ex vivo [75]. It may act by interacting with the regulatory receptor BDCA2 and triggering the downstream inhibitory SYK/MEK-ERK1/2 pathway [76], or by inducing the down expression of TLR9 itself by an unknown mechanism [45].

BDCA3^+^/Clec9A^+^ myeloid DCs [77], which are responsible for IFN-λ production through the activation of TLR3 in particular and represent 10% of total DC in the liver (unpublished data), were also recently shown to be functionally impaired in the blood and liver compartment of CHB patients [69]. HBsAg would also be involved in the inhibition of these cells, as the exposition of naive cells to this antigen recapitulates the inhibitory phenotype. Thus, HBV would be capable of reducing the production of type-I and III IFNs, which are two the main classes of antiviral cytokines, by targeting both subsets of DC and therefore inhibit relevant NK cell activation. 

NK cells are particularly enriched in the liver and are a major source of IFN-γ, a cytokine with direct anti-HBV activity [78,79] and immune-stimulatory properties. They likely play an important role in the resolution of acute HBV infection [39,80,81,82] and, not surprisingly, their impaired functionality in CHB patients, in particular regarding their reduced capacity to produce IFN-γ, has been shown [83,84]. IL-10 and TGF-β present in an HBV-infected liver would be responsible for this inhibition [84]. Regarding their cytotoxic activities in CHB, data are more in favour of an exacerbation of NK-mediated liver damages [85,86]. Interestingly, these features of preserved NK cell cytotoxicity and impaired IFN-γ production are found in both chronic HBV and HCV infections [87], thus pointing toward convergent mechanisms in chronic hepatitis. This complexity of the HBV-driven modulation of NK functions has important implications regarding therapeutic manipulation.

### 2.5. Interplay between HBV and Monocytes/Macrophages (Figure 2)

Resident macrophage cells are thought to play a central role with respect to the peculiarities of liver immune functions [88,89]. They can have opposite functions, according to physiological or pathological settings. They largely contribute to the basal liver immune tolerance, but can also be involved in antiviral responses. They can either hasten recovery after liver injury [90], or be involved in immune-pathogenesis [88,89]. Echoing this complexity, the ontology of human liver resident macrophages, also called KCs, remains a very active field of research and is not well known, as compared to what is known for mice; this is largely due to the lack of accessibility to normal and pathologic human liver, which prevents relevant phenotypic and functional immune studies [88,89]. Phenotypically, it is very difficult to distinguish genuine liver resident macrophages (i.e., long-lived, self-renewing KCs originating from embryonic progenitor) from monocyte-derived macrophages. Indeed, with the onset of inflammation, monocytes are readily recruited to the liver, where they can differentiate into macrophages to replace killed resident ones, thus further complicating analyses [89]. It is therefore not surprising that data on the effect of HBV on macrophages/monocytes and the role of these cells in the natural history of the disease have been generated with sub-optimally characterized immune entities. 

Despite these considerations, it has been shown in mice hydrodynamically injected/transduced with AAV-HBV, that the depletion of liver macrophages/monocytes, or the transduction of TLR2-deficient mice, was associated with a faster clearance of infection via a specific CD8^+^ T-cell response [91]. This suggests a positive proviral role of these TLR2-positive cells for the establishment of a persistent infection in immune-competent mice. This came as a surprise, as these myeloid cells are the main producers of IL-1β, a cytokine bearing strong direct anti-HBV properties [78,92]. However, early contact of HBV with these cells could help prevent the production of this cytokine and promote the production of immune-suppressive ones. Using freshly isolated KCs from human resections, it was indeed shown ex vivo that HBV was capable of blocking the AIM2-inflammasome-mediated production of IL-1β via an HBsAg-mediated mechanism [19]. Moreover, we have shown that HBV can favor an alternative differentiation and promote the production of IL-10 by both HBsAg- and HBc-driven mechanisms, while inhibiting the production of IL-1 or IL-6 (unpublished data). Knowing that KCs in resting livers are already prone to produce IL-10 and TGF-β [93], it may be that HBV further amplifies this phenotype and further reinforces local immune tolerance to promote its early spreading. Related to this, the group of Tian has shown that KCs contributed to HBV-specific T-cell exhaustion in a mouse model, through a core/HBc antigen-TLR2 interaction mechanism [94].

In the context of chronic infection, there is currently no comprehensive study reporting data on the changes in frequency or altered functions of liver KCs/macrophages/monocytes. Again, all available data derive from analyses performed with blood-derived macrophages/monocytes. In this setting, several groups have shown that there was no alteration of the numbers of the different monocyte populations (CD16^−^/CD14^+^) relative to the amount of HBV DNA, HBeAg, and HBsAg [35,65]. In contrast, it was reported that inflammatory CD16^+^ intermediate monocytes are increased in frequency in immune active patients [95], and correlates with ALT elevation, hence suggesting a contribution to liver immune-pathogenesis. The latter was particularly obvious in a double-humanized mouse model, with both a human immune system and human liver cells, in which HBV was capable of inducing a strong immune-pathogenesis, which was mediated by M2-like macrophages. 

To conclude, the interplay between HBV and macrophages/monocytes is rather complex, and this picture is further complicated by conflicting studies performed with poorly characterized immune cells. Better markers for a better phenotyping of these cells are awaited to further investigate the beneficial or detrimental (or both) roles of liver macrophages in HBV natural history. However, as clearly established in cancer biology, it is likely that tumor-associated macrophage (TAM)-like macrophages, endowed with M2-like functions, could play a role in both the maintenance of HBV replication and pathogenesis.

## 3. Interplay between HBV and Innate Immunity: Therapeutic Insights

### 3.1. Can We Improve the Efficacy and Use of IFN-α-Based Therapy?

The past and current clinical use of recombinant IFN-α as an anti-HCV and anti-HBV drug is the best proof of concept that innate immune effectors can be therapeutically valuable, despite their intrinsic low safety profile. The treatment of CHB patients with Peg-IFN-α leads to less than 10% of HBsAg loss (i.e., in a setting of trials; even less in real life), which is currently recognized as a good marker of a functional cure [96]. The reasons why some patients benefit from such a treatment are unclear. Besides virologic and biochemical reasons (i.e., pre-therapeutic viral load, viral genotype, level of pre-therapeutic inflammation), the genetics of the host is likely involved. It would be great to identify biomarkers associated with sustained virologic response (SVR) off-treatment to prevent the unnecessary exposure of CHB patients to Peg-IFN-α, which is responsible for multiple side effects and is not well-tolerated [96].

Virus-related mechanisms of resistance to IFN-α at subcellular levels are currently unknown. It could be that some viral proteins impair the Janus kinase/signal transducers and activators of transcription (JAK/STAT) pathway, as shown in vitro or in liver-humanized mouse models [97,98]. Recently, it was shown that IFN-α could induce, by epigenetic remodeling, the silencing of cccDNA (i.e., inhibition of transcription [99]), which may account for the antiviral effect of the drug in humans, and even in some circumstances, to its degradation via an APOBEC3A-dependent mechanism [100].

If IFN-α administration is meant to boost immunity and help break tolerance to the virus, it has nevertheless been shown that a strong exposure to IFN-α in CHB patients was associated with a NK/NKT-mediated increased killing of HBV-specific T-cells overexpressing the TNF-Related Apoptosis-Inducing Ligand (TRAIL) death receptor [101,102]. As the use of nucleoside analogs (NA) in CHB was associated with a restoration of T-cell functions [103], there was a rationale for combining Peg-IFN-α and a potent second generation NA [104]. The results of a large open-label active-controlled study aiming at determining the benefit of a combination between Peg-IFN-α and Tenofovir, indeed showed that the long-term combination of these two drugs increases the HBsAg loss rate [105], thus encouraging further clinical studies in this respect. In particular, it would be interesting to have biomarkers to identify patients that would benefit the most from IFN-based therapies. In the future, it would be interesting to block the TRAIL-dependent killing of HBV-specific T-cells by monoclonal antibodies, as for other check-point inhibition strategies, to further improve the Peg-IFN-α/NA combination by correcting NK/NKT altered functions [11].

### 3.2. Is There a Place for Other Innate Immune Stimulators?

Beside IFN-α, the use of the less toxic IFN-λ was also proposed to combat viral hepatitis. Indeed, the pattern of expression of the IFN-λ receptor was more restricted to epithelial cells and fewer immune cells, and this IFN has been shown to induce less side effects, as compared to IFN-α. In the clinical trial, it was recently shown that Peg-IFN-λ is as efficient as, but not superior to, Peg-IFN-α for treatment [106]. If further development for CHB is unlikely, the clinical evaluation of Peg-IFN-λ is pursued in the context of co-infection with the hepatitis delta virus. 

Recently, a large body of evidence has shown that many IFNs/cytokines, including IFN-α, IFN-γ, IFN-β, IFN-λ, IL-6, TNF-α, and IL-1β were capable of inhibiting HBV replication in hepatocytes, in the absence of immune cells [78,79,100,107]. These innate effectors are capable of inhibiting the transcription of cccDNA (thought to be through distinct molecular mechanisms; unpublished data), and some of them are also capable of inducing the degradation of cccDNA in vitro, as initially shown with LT-βR agonists, which were particularly potent in this respect [100]. If the development of LT-βR agonists (i.e., monoclonal antibodies and genetically engineered agonists) is going to be pursued, the use of injectable recombinant pro-inflammatory cytokines in humans, other than IFNs, is unlikely. A sound approach to take advantage of the direct antiviral characteristics of these molecules would be to allow their endogenous, local, and timely controlled de novo synthesis in CHB patients. For this, further fundamental studies are needed to determine, in particular, how HBV is capable of blocking their production and/or antiviral effect in vivo. In this respect, works aiming at a better understanding of the mechanism of the blockade of IL-1β production (IL-1β being the most active cytokines tested in vitro [78]), by liver macrophages/monocytes would be instrumental.

### 3.3. Development of PRR Agonists for Combinational Therapeutic Approaches

Currently, no PRR agonists have been approved for the treatment of a chronic infection. Some PRR agonists are currently developed as adjuvant for prophylactic or therapeutic vaccinations in the field of infectiology and oncology. In the HBV field, the antiviral properties of TLR agonists have been described more than ten years ago in HBV-transgenic mice [108]. More recently, we reported another comparative screen of the in vitro activity of TLR and RLR agonists [109]. In this latest screen, TLR2 and TLR3 ligands were shown to be of particular interest, as compared to TLR7 or TLR9 agonists for instance, as they could not only stimulate immune cells, but also have a direct effect in infected hepatocytes, through the activation of IRFs and/or NFκB pathways. The TLR2 ligand Pam3CSK4 was found to be extremely potent (activity in the nM range) when used in primary hepatocytes [109], as compared to its reported efficacy in hepatoma cell models [110]. Interestingly, it was shown in the woodchuck model that a treatment with NA leading to a decrease in Woodchuck hepatitis virus (WHV) viremia was associated with a re-expression of TLR2 in the liver. Hence, a combination between the TLR2 agonist and NA, or other Direct-Acting-Agents (DAAs) developed against HBV, could be a way forward to circumvent the HBV-induced inhibition of TLR2 expression observed in CHB patients [44,111]. An obstacle to the therapeutic development of TLR2 agonist is the fear of a “cytokine storm” that could occur upon systemic administration. The vectorization of TLR2 agonist into nanoparticles, which could be specifically delivered to the liver, could help circumvent this problem and would be useful to study.

TLR9 agonists have a theoretical interest as they are the only ones capable of inducing the formation of iMATEs in a mouse liver, which are a tertiary immune structure composed of myeloid cells providing a niche for the proliferation and maturation of T-cells [112]. Interestingly, the HBV genome contains CpG motifs that have been shown to be either inhibit or activate TLR9 [45], thus suggesting that HBV could naturally modulate the TLR9 pathway. In this respect, it was also shown that TLR9 expression in the Peripheral Blood Mononuclear Cell (PBMC) of infected patients was reduced; the demonstration of this is pending in liver-derived mononuclear cells. This would have consequences on the use of the TLR9 agonist in therapy. However, TLR9 ligands have been successfully encapsulated into nanoparticles and have shown an improved efficacy in boosting prophylactic HBV immunization [113]. This could be helpful to improve the efficacy of TLR9 agonists in vivo, as TLR9 agonists have so far been shown to be less active as compared to those of TLR2 or TLR7 in the woodchuck model [114]. Other PRR agonists, including those working through cGAS and STING [115,116,117], or those working through RIG-I/NOD2 [118], are being investigated for their potential anti-HBV properties.

The current clinical development of TLR7 agonists for CHB is more opportunistic than based on a demonstrated superiority of action on the virus. Indeed, amongst all PRR agonists, TLR7 ligands, which are small heterocyclic molecules, are the only ones to be deliverable orally, which is a tremendous advantage over other ligands. The best-characterized and advanced TLR7-L agonist is GS-9620. This agonist has demonstrated, in mono-therapy, an extremely potent anti-HBV activity in infected chimpanzees and woodchucks, with a very strong, yet unexpected, effect on the cccDNA level in the latest model [119,120]. These results have prompted phase-1b/2 clinical evaluations and official results regarding virologic aspects are awaited. One critical point is related to the doses that have been selected for administration; it is possible that they would end-up being suboptimal, as safety was the main concern of these trials. Mechanistically, GS-9620 works by inducing the production of IFN-α, mainly by pDC, or related mucosal cells, in the upper gut [121,122]. An interesting path forward will be to test this molecule in association with either NA or other DAA (e.g., core assembly inhibitors) in current development, as well as with therapeutic vaccines. Indeed, PRR agonists are mainly meant to have adjacent activity in the context of combination therapy. In this respect, the excellent results obtained in monotherapy in animal models are more indicative than predictive of what will happen in humans.

### 3.4. Manipulation of the Numbers or Biological Activity of Innate Immune Cells with Impaired Functions in CHB Patients to Help Restore HBV Immune Control

Therapeutic concepts, based on processes such a manipulation, are more complex to implement, as we have to keep in mind that immune responses in CHB patients are a subtle equilibrium between the lack of antiviral activity and excessive pro-inflammatory or unspecific killing activities, which could worsen immune-pathogenesis.

Beside unspecific innate immune activation via immune-stimulators (IFN-α or PRR agonist that was discussed above), one could envisage to specifically restore beneficial or inhibit detrimental innate immune cell functions. Myeloid cells endowed with M2-like functions, similar to those of TAMs, are likely to be involved in the persistence of HBV replication. In this respect, MDSC were clearly shown to be associated with a dampening of HBV-specific and bystander T-cell responses in CHB. The depletion (or re-differentiation into antiviral myeloid cells) of such cells or an inhibition of their functions could help to restore the immune function, particularly in low-inflammatory/immune tolerant patients with high viremia, no ALT elevation and, yet, a relevant number of HBV-specific T-cells (as compared to a later stage of the disease). This concept was exemplified by the use of anti-CSF-R1 antibodies to inhibit TAM function in cancer biology [123] and a similar strategy could be implemented by targeting a specific marker of MDSC. On the biochemical side, Tadalafil, a FDA-approved small molecule of the PDE5 inhibitor family, was shown to inhibit the suppressive functions of MDCS in cancer patients, notably by decreasing arginase expression [124]. As for other check-point inhibitors (Ipilimumab, Nivolumab, Pembrolizumab), the repositioning of these drugs to chronic infections is only a matter of time and carefully implemented clinical trials. 

If the stimulation of immune cells capable of locally and temporally producing either IFN-α (via pDC), IFNs-λ (via BDCA3^+^ cells) or IL-1β (via myeloid cells), which have direct anti-HBV properties [107], which is a sound approach theoretically, and could be achieved for instance by PRR agonists, a cell-therapy based on pDC or BDCA3^+^ DC loaded with HBV-derived peptides could also be an interesting approach. Indeed, these cells could both produce cytokines and elicit T-cell functions via their APC properties. A proof-of-concept study was recently reported in the context of HBV, by Martinet and colleagues [125].

## 4. Conclusions

In the last 10 years, our understanding of the interplay between HBV and innate immune functions has greatly improved and opened novel avenues of research, in particular regarding the potential use of immune-therapeutic components in the setting of expected combination therapies. However, due to the anticipated difficulties in terms of implementation, more fundamental and translational researches are needed to pave this long way to success. In contrast to what has been achieved in the field of HCV, where the sole use of DAAs has led to viral eradication, it is likely that the restoration of immune control of HBV replication, by means of Host-Targeting-Agents (HTA), will be instrumental to achieve a functional cure in CHB patients. In this respect, an IFN-α based regimen will likely not disappear from the therapeutic landscape in the near future. Hence, current clinical investigations with novel DAA/HTA do contain therapeutic arms featuring a combination with Peg-IFN-α. This combinational rule also holds true for the potential development of other immune-stimulators, including PRR agonists or any other strategy to restore the transient production of antiviral cytokines. 

One awaited demonstration is whether a therapeutically induced drop of HBsAg antigenemia (by whatever means) would help restore endogenous immune functions. Indeed, mechanistically speaking, it seems that HBsAg is often involved in HBV-driven immune subversion. One could think that its loss would be a step toward recovered immune functions and the in vivo demonstration of this theory is eagerly awaited. Another viral target, which could be of interest to help restore the immune response by other means, is HBc. If underlying mechanisms are still to be defined, a combination of the TLR7 agonist or Peg-IFN with core/capsid/HBc assembly inhibitors seems to synergistically improve HBsAg loss in the AAV-HBV immune competent mouse model [126,127] (see also patent number: WO-2016146598), thus warranting further clinical studies in humans.

## References

[1] Crispe I.N. (2009). The Liver as a Lymphoid Organ. Annu. Rev. Immunol..

[2] Crispe I.N. (2011). Liver antigen-presenting cells. J. Hepatol..

[3] Protzer U., Maini M.K., Knolle P.A. (2012). Living in the liver: Hepatic infections. Nat. Rev. Immunol..

[4] Guidotti L.G., Chisari F.V. (2006). Immunobiology and pathogenesis of viral hepatitis. Annu. Rev. Pathol..

[5] Heymann F., Tacke F. (2016). Immunology in the liver—From homeostasis to disease. Nat. Rev. Gastroenterol. Hepatol..

[6] Knolle P.A., Thimme R. (2014). Hepatic immune regulation and its involvement in viral hepatitis infection. Gastroenterology.

[7] Crispe I.N. (2014). Immune tolerance in liver disease. Hepatology.

[8] Bertoletti A., Ferrari C. (2016). Adaptive immunity in HBV infection. J. Hepatol..

[9] Park S.H., Rehermann B. (2014). Immune responses to HCV and other hepatitis viruses. Immunity.

[10] Bertoletti A., Ferrari C. (2012). Innate and adaptive immune responses in chronic hepatitis B virus infections: Towards restoration of immune control of viral infection. Gut.

[11] Maini M.K., Gehring A.J. (2016). The role of innate immunity in the immunopathology and treatment of HBV infection. J. Hepatol..

[12] Pandey S., Kawai T., Akira S. (2014). Microbial sensing by Toll-like receptors and intracellular nucleic acid sensors. Cold Spring Harb. Perspect. Biol..

[13] Broz P., Dixit V.M. (2016). Inflammasomes: Mechanism of assembly, regulation and signalling. Nat. Rev. Immunol..

[14] Crowley S.M., Vallance B.A., Knodler L.A. (2017). Noncanonical inflammasomes: Antimicrobial defense that does not play by the rules. Cell. Microbiol..

[15] Crispe I.N. (2016). Hepatocytes as Immunological Agents. J. Immunol..

[16] Luangsay S., Ait-Goughoulte M., Michelet M., Floriot O., Bonnin M., Gruffaz M., Rivoire M., Fletcher S., Javanbakht H., Lucifora J. (2015). Expression and functionality of Toll- and RIG-like receptors in HepaRG cells. J. Hepatol..

[17] Vegna S., Gregoire D., Moreau M., Lassus P., Durantel D., Assenat E., Hibner U., Simonin Y. (2016). NOD1 Participates in the Innate Immune Response Triggered by Hepatitis C Virus Polymerase. J. Virol..

[18] Thomsen M.K., Nandakumar R., Stadler D., Malo A., Valls R.M., Wang F., Reinert L.S., Dagnaes-Hansen F., Hollensen AK., Mikkelsen J.G. (2016). Lack of immunological DNA sensing in hepatocytes facilitates hepatitis B virus infection. Hepatology.

[19] Zannetti C., Roblot G., Charrier E., Ainouze M., Tout I., Briat F., Isorce F., Faure-Dupuy S., Michelet M., Marotel M. (2016). Characterization of the Inflammasome in Human Kupffer Cells in Response to Synthetic Agonists and Pathogens. J. Immunol..

[20] Gripon P., Rumin S., Urban S., Le Seyec J., Glaise D., Cannie I., Guyomard C., Lucas J., Trepo C., Guguen-Guillouzo C. (2002). Infection of a human hepatoma cell line by hepatitis B virus. Proc. Natl. Acad. Sci. USA.

[21] Sells M.A., Chen M.L., Acs G. (1987). Production of hepatitis B virus particles in Hep G2 cells transfected with cloned hepatitis B virus DNA. Proc. Natl. Acad. Sci. USA.

[22] Ladner S.K., Otto M.J., Barker C.S., Zaifert K., Wang G.H., Guo J.T., Seeger C., King R.W. (1997). Inducible expression of human hepatitis B virus (HBV) in stably transfected hepatoblastoma cells: A novel system for screening potential inhibitors of HBV replication. Antimicrob. Agents Chemother..

[23] Gripon P., Diot C., Thézé N., Fourel I., Loreal O., Brechot C., Guguen-Guillouzo C. (1988). Hepatitis B virus infection of adult human hepatocytes cultured in the presence of dimethyl sulfoxide. J. Virol..

[24] Li W., Urban S. (2016). Entry of hepatitis B and hepatitis D virus into hepatocytes: Basic insights and clinical implications. J. Hepatol..

[25] Luangsay S., Gruffaz M., Isorce N., Testoni B., Michelet M., Faure-Dupuy S., Maadadi S., Ait-Goughoulte M., Parent R., Rivoire M. (2015). Early inhibition of hepatocyte innate responses by hepatitis B virus. J. Hepatol..

[26] Vanlandschoot P., Van Houtte F., Serruys B., Leroux-Roels G. (2007). Contamination of a recombinant hepatitis B virus nucleocapsid preparation with a human B-cell activator. J. Virol..

[27] Cooper A., Tal G., Lider O., Shaul Y. (2005). Cytokine induction by the hepatitis B virus capsid in macrophages is facilitated by membrane heparan sulfate and involves TLR2. J. Immunol..

[28] Huang Z., Ge J., Pang J., Liu H., Chen J., Liao B., Huang X., Zuo D., Sun J., Lu M. (2015). Aberrant expression and dysfunction of TLR2 and its soluble form in chronic HBV infection and its regulation by antiviral therapy. Antivir. Res..

[29] Yoneda M., Hyun J., Jakubski S., Saito S., Nakajima A., Schiff E.R., Thomas E. (2016). Hepatitis B Virus and DNA Stimulation Trigger a Rapid Innate Immune Response through NF-kappaB. J. Immunol..

[30] Seeger C., Zoulim F., Mason W.S., Knipe D.M., Howley P.M. (2015). Hepadnaviruses. Field’s Virology.

[31] Sato S., Li K., Kameyama T., Hayashi T., Ishida Y., Murakami S., Watanabe T., Iijima S., Sakurai Y., Watashi K. (2015). The RNA sensor RIG-I dually functions as an innate sensor and direct antiviral factor for hepatitis B virus. Immunity.

[32] Riviere L., Gerossier L., Ducroux A., Dion S., Deng Q., Michel M.L., Buendia M.A., Hantz O., Neuveut C. (2015). HBx relieves chromatin-mediated transcriptional repression of hepatitis B viral cccDNA involving SETDB1 histone methyltransferase. J. Hepatol..

[33] Decorsiere A., Mueller H., van Breugel P.C., Abdul F., Gerossier L., Beran R.K., Livingston C.M., Niu C., Fletcher S.P., Hantz O. (2016). Hepatitis B virus X protein identifies the Smc5/6 complex as a host restriction factor. Nature.

[34] Vanlandschoot P., Van Houtte F., Roobrouck A., Farhoudi A., Stelter F., Peterson D.L., Gomez-Gutierrez J., Gavilanes F., Leroux-Roels G. (2002). LPS-binding protein and CD14-dependent attachment of hepatitis B surface antigen to monocytes is determined by the phospholipid moiety of the particles. J. Gen. Virol..

[35] Gehring A.J., Haniffa M., Kennedy P.T., Ho Z.Z., Boni C., Shin A., Banu N., Chia A., Lim S.G., Ferrari C. (2013). Mobilizing monocytes to cross-present circulating viral antigen in chronic infection. J. Clin. Investig..

[36] Van Montfoort N., van der Aa E., van den Bosch A., Brouwers H., Vanwolleghem T., Janssen H.L., Javanbakht H., Buschow S.I., Woltman A.M. (2016). Hepatitis B Virus Surface Antigen Activates Myeloid Dendritic Cells via a Soluble CD14-Dependent Mechanism. J. Virol..

[37] Wieland S., Thimme R., Purcell R.H., Chisari F.V. (2004). Genomic analysis of the host response to hepatitis B virus infection. Proc. Natl. Acad. Sci. USA.

[38] Stacey A.R., Norris P.J., Qin L., Haygreen E.A., Taylor E., Heitman J., Lebedeva M., DeCamp A., Li D., Grove D. (2009). Induction of a striking systemic cytokine cascade prior to peak viremia in acute human immunodeficiency virus type 1 infection, in contrast to more modest and delayed responses in acute hepatitis B and C virus infections. J. Virol..

[39] Dunn C., Peppa D., Khanna P., Nebbia G., Jones M., Brendish N., Lascar R.M., Brown D., Gilson R.J., Tedder R.J. (2009). Temporal analysis of early immune responses in patients with acute hepatitis B virus infection. Gastroenterology.

[40] Das A., Ellis G., Pallant C., Lopes A.R., Khanna P., Peppa D., Chen A., Blair P., Dusheiko G., Gill U. (2012). IL-10-producing regulatory B cells in the pathogenesis of chronic hepatitis B virus infection. J. Immunol..

[41] Hosel M., Quasdorff M., Wiegmann K., Webb D., Zedler U., Broxtermann M., Tedjokusumo R., Esser K., Arzberger S., Kirschning C.J. (2009). Not interferon, but interleukin-6 controls early gene expression in hepatitis B virus infection. Hepatology.

[42] Shlomai A., Schwartz R.E., Ramanan V., Bhatta A., de Jong Y.P., Bhatia S.N., Rice C.M. (2014). Modeling host interactions with hepatitis B virus using primary and induced pluripotent stem cell-derived hepatocellular systems. Proc. Natl. Acad. Sci. USA.

[43] Lebosse F., Testoni B., Fresquet J., Facchetti F., Galmozzi E., Fournier M., Hervieu V., Berthillon P., Berby F., Bordes I. (2016). Intrahepatic innate immune response pathways are downregulated in untreated chronic hepatitis B patients. J. Hepatol..

[44] Visvanathan K., Skinner N.A., Thompson A.J., Riordan S.M., Sozzi V., Edwards R., Rodgers S., Kurtovic J., Chang J., Lewin S. (2007). Regulation of Toll-like receptor-2 expression in chronic hepatitis B by the precore protein. Hepatology.

[45] Vincent I.E., Zannetti C., Lucifora J., Norder H., Protzer U., Hainaut P., Zoulim F., Tommasino M., Trépo C., Hasan U. (2011). Hepatitis B virus impairs TLR9 expression and function in plasmacytoid dendritic cells. PLoS ONE.

[46] Fletcher S.P., Chin D.J., Ji Y., Iniguez A.L., Taillon B., Swinney D.C., Ravindran P., Cheng D.T., Bitter H., Lopatin U. (2012). Transcriptomic analysis of the woodchuck model of chronic hepatitis B. Hepatology.

[47] Giersch K., Allweiss L., Volz T., Helbig M., Bierwolf J., Lohse A.W., Pollok J.M., Petersen J., Dandri M., Lütgehetmann M. (2015). Hepatitis Delta co-infection in humanized mice leads to pronounced induction of innate immune responses in comparison to HBV mono-infection. J. Hepatol..

[48] Alfaiate D., Lucifora J., Abeywickrama-Samarakoon N., Michelet M., Testoni B., Cortay J.C., Sureau C., Zoulim F., Dény P., Durantel D. (2016). HDV RNA replication is associated with HBV repression and interferon-stimulated genes induction in super-infected hepatocytes. Antivir. Res..

[49] Wu J., Meng Z., Jiang M., Pei R., Trippler M., Broering R., Bucchi A., Sowa J.P., Dittmer U., Yang D. (2009). Hepatitis B virus suppresses toll-like receptor-mediated innate immune responses in murine parenchymal and nonparenchymal liver cells. Hepatology.

[50] Gruffaz M., Testoni B., Luangsay S., Fusil F., Malika A.G., Mancip J., Petit M., Javanbakht H., Cosset F.L., Zoulim F. (2013). The nuclear function of Hepatitis B capsid (HBc) protein is to inhibit IFN response very early after infection of hepatocytes. Hepatology.

[51] Bock C.T., Schwinn S., Locarnini S., Fyfe J., Manns M.P., Trautwein C., Zentgraf H. (2001). Structural organization of the hepatitis B virus minichromosome. J. Mol. Biol..

[52] Fernandez M., Quiroga J.A., Carreno V. (2003). Hepatitis B virus downregulates the human interferon-inducible MxA promoter through direct interaction of precore/core proteins. J. Gen. Virol..

[53] Guo Y.H., Li Y.N., Zhao J.R., Zhang J., Yan Z. (2011). HBc binds to the CpG islands of HBV cccDNA and promotes an epigenetic permissive state. Epigenetics.

[54] Yi Z., Chen J., Kozlowski M., Yuan Z. (2015). Innate detection of hepatitis B and C virus and viral inhibition of the response. Cell. Microbiol..

[55] Jiang M., Broering R., Trippler M., Poggenpohl L., Fiedler M., Gerken G., Lu M., Schlaak J.F. (2014). Toll-like receptor-mediated immune responses are attenuated in the presence of high levels of hepatitis B virus surface antigen. J. Viral Hepat..

[56] Lang T., Lo C., Skinner N., Locarnini S., Visvanathan K., Mansell A. (2011). The hepatitis B e antigen (HBeAg) targets and suppresses activation of the toll-like receptor signaling pathway. J. Hepatol..

[57] Wang S., Chen Z., Hu C., Qian F., Cheng Y., Wu M., Shi B., Chen J., Hu Y., Yuan Z. (2013). Hepatitis B virus surface antigen selectively inhibits TLR2 ligand-induced IL-12 production in monocytes/macrophages by interfering with JNK activation. J. Immunol..

[58] Hong Y., Zhou L., Xie H., Zheng S. (2015). Innate immune evasion by hepatitis B virus-mediated downregulation of TRIF. Biochem. Biophys. Res. Commun..

[59] Jiang J., Tang H. (2010). Mechanism of inhibiting type I interferon induction by hepatitis B virus X protein. Protein Cell.

[60] Kumar M., Jung S.Y., Hodgson A.J., Madden C.R., Qin J., Slagle B.L. (2011). Hepatitis B virus regulatory HBx protein binds to adaptor protein IPS-1 and inhibits the activation of beta interferon. J. Virol..

[61] Wei C., Ni C., Song T., Liu Y., Yang X., Zheng Z., Jia Y., Yuan Y., Guan K., Xu Y. (2010). The hepatitis B virus X protein disrupts innate immunity by downregulating mitochondrial antiviral signaling protein. J. Immunol..

[62] Slagle B.L., Andrisani O.M., Bouchard M.J., Lee C.G., Ou J.H., Siddiqui A. (2015). Technical standards for hepatitis B virus X protein (HBx) research. Hepatology.

[63] Liu Y., Li J., Chen J., Li Y., Wang W., Du X., Song W., Zhang W., Lin L., Yuan Z. (2015). Hepatitis B virus polymerase disrupts K63-linked ubiquitination of STING to block innate cytosolic DNA-sensing pathways. J. Virol..

[64] Wang H., Ryu W.S. (2010). Hepatitis B virus polymerase blocks pattern recognition receptor signaling via interaction with DDX3: Implications for immune evasion. PLoS Pathog.

[65] Boltjes A., Groothuismink Z.M., van Oord G.W., Janssen H.L., Woltman A.M., Boonstra A. (2014). Monocytes from chronic HBV patients react in vitro to HBsAg and TLR by producing cytokines irrespective of stage of disease. PLoS ONE.

[66] De Groen R.A., Hou J., van Oord G.W., Groothuismink Z.M., van der Heide M., de Knegt R.J., Boonstra A. (2017). NK cell phenotypic and functional shifts coincide with specific clinical phases in the natural history of chronic HBV infection. Antivir. Res..

[67] Martinet J., Dufeu-Duchesne T., Bruder Costa J., Larrat S., Marlu A., Leroy V., Plumas J., Aspord C. (2012). Altered functions of plasmacytoid dendritic cells and reduced cytolytic activity of natural killer cells in patients with chronic HBV infection. Gastroenterology.

[68] Tjwa E.T., Zoutendijk R., van Oord G.W., Boeijen L.L., Reijnders J.G., van Campenhout M.J., de Knegt R.J., Janssen H.L., Woltman A.M., Boonstra A. (2016). Similar frequencies, phenotype and activation status of intrahepatic NK cells in chronic HBV patients after long-term treatment with tenofovir disoproxil fumarate (TDF). Antivir. Res..

[69] Van der Aa E., Buschow S.I., Biesta P.J., Janssen H.L., Woltman A.M. (2016). The effect of chronic hepatitis B virus infection on BDCA3+ dendritic cell frequency and function. PLoS ONE.

[70] Pallett L.J., Gill U.S., Quaglia A., Sinclair L.V., Jover-Cobos M., Schurich A., Singh K.P., Thomas N., Das A., Chen A. (2015). Metabolic regulation of hepatitis B immunopathology by myeloid-derived suppressor cells. Nat. Med..

[71] Fang Z., Li J., Yu X., Zhang D., Ren G., Shi B., Wang C., Kosinska A.D., Wang S., Zhou X. (2015). Polarization of monocytic myeloid-derived suppressor cells by hepatitis B surface antigen is mediated via ERK/IL-6/STAT3 signaling feedback and restrains the activation of T-cells in chronic hepatitis B virus infection. J. Immunol..

[72] Swiecki M., Colonna M. (2015). The multifaceted biology of plasmacytoid dendritic cells. Nat. Rev. Immunol..

[73] Woltman A.M., Op den Brouw M.L., Biesta P.J., Shi C.C., Janssen H.L. (2011). Hepatitis B virus lacks immune activating capacity, but actively inhibits plasmacytoid dendritic cell function. PLoS ONE.

[74] Hong J., Gong Z.J. (2008). Human plasmacytoid dendritic cells from patients with chronic hepatitis B virus infection induce the generation of a higher proportion of CD4(+) and CD25(+) regulatory T-cells compared with healthy patients. Hepatol. Res..

[75] Xu Y., Hu Y., Shi B., Zhang X., Wang J., Zhang Z., Shen F., Zhang Q., Sun S., Yuan Z. (2009). Hbsag inhibits TLR9-mediated activation and IFN-alpha production in plasmacytoid dendritic cells. Mol. Immunol..

[76] Gilliet M., Cao W., Liu Y.J. (2008). Plasmacytoid dendritic cells: Sensing nucleic acids in viral infection and autoimmune diseases. Nat. Rev. Immunol..

[77] Van der Aa E., van Montfoort N., Woltman A.M. (2015). BDCA3(+)CLEC9a(+) human dendritic cell function and development. Semin. Cell Dev. Biol..

[78] Isorce N., Testoni B., Locatelli M., Fresquet J., Rivoire M., Luangsay S., Zoulim F., Durantel D. (2016). Antiviral activity of various interferons and pro-inflammatory cytokines in non-transformed cultured hepatocytes infected with hepatitis B virus. Antivir. Res..

[79] Xia Y., Stadler D., Lucifora J., Reisinger F., Webb D., Hosel M., Michler T., Wisskirchen K., Cheng X., Zhang K. (2016). Interferon-gamma and tumor necrosis factor-alpha produced by T-cells reduce the HBV persistence form, cccDNA, without cytolysis. Gastroenterology.

[80] Fisicaro P., Valdatta C., Boni C., Massari M., Mori C., Zerbini A., Orlandini A., Sacchelli L., Missale G., Ferrari C. (2009). Early kinetics of innate and adaptive immune responses during hepatitis B virus infection. Gut.

[81] Guy C.S., Mulrooney-Cousins P.M., Churchill N.D., Michalak T.I. (2008). Intrahepatic expression of genes affiliated with innate and adaptive immune responses immediately after invasion and during acute infection with woodchuck hepadnavirus. J. Virol..

[82] Webster G.J., Reignat S., Maini M.K., Whalley S.A., Ogg G.S., King A., Brown D., Amlot P.L., Williams R., Vergani D. (2000). Incubation phase of acute hepatitis B in man: Dynamic of cellular immune mechanisms. Hepatology.

[83] Oliviero B., Varchetta S., Paudice E., Michelone G., Zaramella M., Mavilio D., De Filippi F., Bruno S., Mondelli M.U. (2009). Natural killer cell functional dichotomy in chronic hepatitis B and chronic hepatitis C virus infections. Gastroenterology.

[84] Peppa D., Micco L., Javaid A., Kennedy P.T., Schurich A., Dunn C., Pallant C., Ellis G., Khanna P., Dusheiko G. (2010). Blockade of immunosuppressive cytokines restores NK cell antiviral function in chronic hepatitis B virus infection. PLoS Pathog.

[85] Okazaki A., Hiraga N., Imamura M., Hayes C.N., Tsuge M., Takahashi S., Aikata H., Abe H., Miki D., Ochi H. (2012). Severe necroinflammatory reaction caused by natural killer cell-mediated Fas/Fas ligand interaction and dendritic cells in human hepatocyte chimeric mouse. Hepatology.

[86] Zhang Z., Zhang S., Zou Z., Shi J., Zhao J., Fan R., Qin E., Li B., Li Z., Xu X. (2011). Hypercytolytic activity of hepatic natural killer cells correlates with liver injury in chronic hepatitis B patients. Hepatology.

[87] Rehermann B. (2015). Natural killer cells in Viral Hepatitis. Cell. Mol. Gastroenterol. Hepatol..

[88] Boltjes A., Movita D., Boonstra A., Woltman A.M. (2014). The role of Kupffer cells in hepatitis B and hepatitis C virus infections. J. Hepatol..

[89] Brempelis K.J., Crispe I.N. (2016). Infiltrating monocytes in liver injury and repair. Clin. Transl. Immunol..

[90] Sitia G., Iannacone M., Aiolfi R., Isogawa M., van Rooijen N., Scozzesi C., Bianchi M.E., von Andrian U.H., Chisari F.V., Guidotti L.G. (2011). Kupffer cells hasten resolution of liver immunopathology in mouse models of viral hepatitis. PLoS Pathog.

[91] Xu L., Yin W., Sun R., Wei H., Tian Z. (2014). Kupffer cell-derived IL-10 plays a key role in maintaining humoral immune tolerance in hepatitis B virus-persistent mice. Hepatology.

[92] Watashi K., Liang G., Iwamoto M., Marusawa H., Uchida N., Daito T., Kitamura K., Muramatsu M., Ohashi H., Kiyohara T. (2013). Interleukin-1 and tumor necrosis factor-alpha trigger restriction of hepatitis B virus infection via a cytidine deaminase activation-induced cytidine deaminase (AID). J. Biol. Chem..

[93] Heymann F., Peusquens J., Ludwig-Portugall I., Kohlhepp M., Ergen C., Niemietz P., Martin C., van Rooijen N., Ochando J.C., Randolph G.J. (2015). Liver inflammation abrogates immunological tolerance induced by Kupffer cells. Hepatology.

[94] Li M., Sun R., Xu L., Yin W., Chen Y., Zheng X., Lian Z., Wei H., Tian Z. (2015). Kupffer cells support hepatitis B virus-mediated CD8+ T-cell exhaustion via Hepatitis B Core Antigen-TLR2 interactions in mice. J. Immunol..

[95] Zhang J.Y., Zou Z.S., Huang A., Zhang Z., Fu J.L., Xu X.S., Chen L.M., Li B.S., Wang F.S. (2011). Hyper-activated pro-inflammatory CD16 monocytes correlate with the severity of liver injury and fibrosis in patients with chronic hepatitis B. PLoS ONE.

[96] Konerman M.A., Lok A.S. (2016). Interferon treatment for Hepatitis B. Clin. Liver Dis..

[97] Christen V., Duong F., Bernsmeier C., Sun D., Nassal M., Heim M.H. (2007). Inhibition of alpha interferon signaling by hepatitis B virus. J. Virol..

[98] Lutgehetmann M., Bornscheuer T., Volz T., Allweiss L., Bockmann J.H., Pollok J.M., Lohse A.W., Petersen J., Dandri M. (2011). Hepatitis B virus limits response of human hepatocytes to interferon-alpha in chimeric mice. Gastroenterology.

[99] Belloni L., Allweiss L., Guerrieri F., Pediconi N., Volz T., Pollicino T., Petersen J., Raimondo G., Dandri M., Levrero M. (2012). Ifn-alpha inhibits HBV transcription and replication in cell culture and in humanized mice by targeting the epigenetic regulation of the nuclear cccdna minichromosome. J. Clin. Investig..

[100] Lucifora J., Xia Y., Reisinger F., Zhang K., Stadler D., Cheng X. (2014). Specific and nonhepatotoxic degradation of nuclear hepatitis B virus cccDNA. Science.

[101] Micco L., Peppa D., Loggi E., Schurich A., Jefferson L., Cursaro C., Panno A.M., Bernardi M., Brander C., Bihl F. (2013). Differential boosting of innate and adaptive antiviral responses during pegylated-interferon-alpha therapy of chronic hepatitis B. J. Hepatol..

[102] Penna A., Laccabue D., Libri I., Giuberti T., Schivazappa S., Alfieri A., Mori C., Canetti D., Lampertico P., Vigano M. (2012). Peginterferon-alpha does not improve early peripheral blood HBV-specific T-cell responses in HBeAg-negative chronic hepatitis. J. Hepatol..

[103] Boni C., Laccabue D., Lampertico P., Giuberti T., Vigano M., Schivazappa S., Alfieri A., Pesci M., Gaeta G.B., Brancaccio G. (2012). Restored function of HBV-specific T-cells after long-term effective therapy with nucleos(t)ide analogues. Gastroenterology.

[104] Thimme R., Dandri M. (2013). Dissecting the divergent effects of interferon-alpha on immune cells: Time to rethink combination therapy in chronic hepatitis B?. J. Hepatol..

[105] Marcellin P., Ahn S.H., Ma X., Caruntu F.A., Tak W.Y., Elkashab M., Chuang W.L., Lim S.G., Tabak F., Mehta R. (2016). Combination of tenofovir disoproxil fumarate and peginterferon alpha-2a increases loss of hepatitis B surface antigen in patients with chronic hepatitis B. Gastroenterology.

[106] Chan H.L., Ahn S.H., Chang T.T., Peng C.Y., Wong D., Coffin C.S., Lim S.G., Chen P.J., Janssen H.L., Marcellin P. (2016). Peginterferon lambda for the treatment of HBeAg-positive chronic hepatitis B: A randomized phase 2b study (LIRA-B). J. Hepatol..

[107] Isorce N., Lucifora J., Zoulim F., Durantel D. (2015). Immune-modulators to combat hepatitis B virus infection: From INF-alpha to novel investigational immunotherapeutic strategies. Antiviral Res..

[108] Isogawa M., Robek M.D., Furuichi Y., Chisari F.V. (2005). Toll-like receptor signaling inhibits hepatitis B virus replication in vivo. J. Virol..

[109] Lucifora J., Maadadi S., Floriot O., Daffis S., Fletcher S., Zoulim F., Durantel D. (2015). Direct antiviral effects of various pattern recognition receptor (PRR) agonists in HBV-replicating hepatocytes. J. Hepatol..

[110] Zhang X., Ma Z., Liu H., Liu J., Meng Z., Broering R., Yang D., Schlaak J.F., Roggendorf M., Lu M. (2012). Role of toll-like receptor 2 in the immune response against hepadnaviral infection. J. Hepatol..

[111] Durantel D., Zoulim F. (2012). Interplay between hepatitis B virus and TLR2-mediated innate immune responses: Can restoration of TLR2 functions be a new therapeutic option?. J. Hepatol..

[112] Huang L.R., Wohlleber D., Reisinger F., Jenne C.N., Cheng R.L., Abdullah Z., Schildberg F.A., Odenthal M., Dienes H.P., van Rooijen N. (2013). Intrahepatic myeloid-cell aggregates enable local proliferation of CD8(+) T-cells and successful immunotherapy against chronic viral liver infection. Nat. Immunol..

[113] Lv S., Wang J., Dou S., Yang X., Ni X., Sun R., Tian Z., Wei H. (2014). Nanoparticles encapsulating hepatitis B virus cytosine-phosphate-guanosine induce therapeutic immunity against HBV infection. Hepatology.

[114] Meng Z., Zhang X., Pei R., Zhang E., Kemper T., Vollmer J., Davis H.L., Glebe D., Gerlich W., Roggendorf M. (2016). Combination therapy including CpG oligodeoxynucleotides and entecavir induces early viral response and enhanced inhibition of viral replication in a woodchuck model of chronic hepadnaviral infection. Antivir. Res..

[115] Dansako H., Ueda Y., Okumura N., Satoh S., Sugiyama M., Mizokami M., Ikeda M., Kato N. (2016). The cyclic GMP-AMP synthetase-STING signaling pathway is required for both the innate immune response against HBV and the suppression of HBV assembly. FEBS J..

[116] Guo F., Han Y., Zhao X., Wang J., Liu F., Xu C., Wei L., Jiang J.D., Block T.M., Guo J.T. (2015). STING agonists induce an innate antiviral immune response against hepatitis B virus. Antimicrob. Agents Chemother..

[117] He J., Hao R., Liu D., Liu X., Wu S., Guo S., Wang Y., Tien P., Guo D. (2016). Inhibition of hepatitis B virus replication by activation of the cGAS-STING pathway. J. Gen. Virol..

[118] Korolowicz K.E., Iyer R.P., Czerwinski S., Suresh M., Yang J., Padmanabhan S., Sheri A., Pandey R.K., Skell J., Marquis J.K. (2016). Antiviral efficacy and host innate immunity associated with SB 9200 treatment in the woodchuck model of chronic hepatitis B. PLoS ONE.

[119] Lanford R.E., Guerra B., Chavez D., Giavedoni L., Hodara V.L., Brasky K.M., Fosdick A., Frey C.R., Zheng J., Wolfgang G. (2013). GS-9620, an oral agonist of Toll-Like receptor-7, induces prolonged suppression of hepatitis B virus in chronically infected chimpanzees. Gastroenterology.

[120] Menne S., Tumas D.B., Liu K.H., Thampi L., AlDeghaither D., Baldwin B.H., Bellezza C.A., Cote P.J., Zheng J., Halcomb R. (2015). Sustained efficacy and seroconversion with the Toll-Like receptor 7 agonist GS-9620 in the woodchuck model of chronic hepatitis B. J. Hepatol..

[121] Gane E.J., Lim Y.-S., Gordon S.C., Visvanathan K., Sicard E., Fedorak R.N., Roberts S., Massetto B., Ye Z., Pflanz S. (2015). The oral Toll-Like receptor-7 agonist GS-9620 in patients with chronic hepatitis B virus infection. J. Hepatol..

[122] Lawitz E., Gruener D., Marbury T., Hill J., Webster L., Hassman D., Nguyen A.H., Pflanz S., Mogalian E., Gaggar A. (2015). Safety, pharmacokinetics and pharmacodynamics of the oral Toll-Like receptor 7 agonist GS-9620 in treatment-naive patients with chronic hepatitis C. Antivir. Ther..

[123] Ries C.H., Cannarile M.A., Hoves S., Benz J., Wartha K., Runza V., Rey-Giraud F., Pradel L.P., Feuerhake F., Klaman I. (2014). Targeting tumor-associated macrophages with anti-CSF-1R antibody reveals a strategy for cancer therapy. Cancer Cell.

[124] Tobin R.P., Davis D., Jordan K.R., McCarter M.D. (2017). The clinical evidence for targeting human myeloid-derived suppressor cells in cancer patients. J. Leukoc. Biol..

[125] Martinet J., Leroy V., Dufeu-Duchesne T., Larrat S., Richard M.J., Zoulim F., Plumas J., Aspord C. (2012). Plasmacytoid dendritic cells induce efficient stimulation of antiviral immunity in the context of chronic hepatitis B virus infection. Hepatology.

[126] Lee A.C., Dhillon A.P., Reid S.P., Thi E.P., Phelps J.R., McClintock K., Li A.H., Pasetka C., Cobarrubias K.D., Majeski S. (2016). Exploring combination therapy for curing HBV: Preclinical studies with capsid inhibitor AB-423 and a sirna agent, ABR-1740. Hepatology.

[127] Mani N., Cole A.G., Ardzinski A., Cai D.W., Cuconati A., Dorsey B.D., Guo F., Guo H.T., Guo J.T., Kultgen S. (2016). The HBV capsid inhibitor AB-423 exhibits a dual mode of action and displays additive/synergistic effects in in vitro combination studies. Hepatology.

